# Honokiol Suppresses Perineural Invasion of Pancreatic Cancer by Inhibiting SMAD2/3 Signaling

**DOI:** 10.3389/fonc.2021.728583

**Published:** 2021-10-04

**Authors:** Tao Qin, Jie Li, Ying Xiao, Xueni Wang, Mengyuan Gong, Qiqi Wang, Zeen Zhu, Simei Zhang, Wunai Zhang, Fang Cao, Liang Han, Zheng Wang, Qingyong Ma, Huanchen Sha

**Affiliations:** ^1^ Department of Hepatobiliary Surgery, The First Affiliated Hospital of Xi’an Jiaotong University, Xi’an, China; ^2^ Center for Translational Medicine, The First Affiliated Hospital of Xi’an Jiaotong University, Xi’an, China; ^3^ Centre for Pancreatic Diseases of Xi’an Jiaotong University, Xi’an, China; ^4^ Key Laboratory of Environment and Genes Related to Diseases, Xi’an Jiaotong University, Xi’an, China

**Keywords:** Honokiol (CID: 72303), perineural invasion (PNI), pancreatic cancer, SMAD2/3, EMT - epithelial to mesenchymal transformation

## Abstract

**Background:**

Perineural invasion (PNI) is an important pathologic feature of pancreatic cancer, and the incidence of PNI in pancreatic cancer is 70%-100%. PNI is associated with poor outcome, metastasis, and recurrence in pancreatic cancer patients. There are very few treatments for PNI in pancreatic cancer. Honokiol (HNK) is a natural product that is mainly obtained from Magnolia species and has been indicated to have anticancer activity. HNK also has potent neurotrophic activity and may be effective for suppressing PNI. However, the potential role of HNK in the treatment of PNI in pancreatic cancer has not been elucidated.

**Methods:**

In our study, pancreatic cancer cells were treated with vehicle or HNK, and the invasion and migration capacities were assessed by wound scratch assays and Transwell assays. A cancer cell-dorsal root ganglion coculture model was established to evaluate the effect of HNK on the PNI of pancreatic cancer. Western blotting was used to detect markers of EMT and neurotrophic factors in pancreatic tissue. Recombinant TGF-β1 was used to activate SMAD2/3 to verify the effect of HNK on SMAD2/3 and neurotrophic factors. The subcutaneous tumor model and the sciatic nerve invasion model, which were established in transgenic engineered mice harboring spontaneous pancreatic cancer, were used to investigate the mechanism by which HNK inhibits EMT and PNI *in vivo*.

**Results:**

We found that HNK can inhibit the invasion and migration of pancreatic cancer cells. More importantly, HNK can inhibit the PNI of pancreatic cancer. The HNK-mediated suppression of pancreatic cancer PNI was partially mediated by inhibition of SMAD2/3 phosphorylation. In addition, the inhibitory effect of HNK on PNI can be reversed by activating SMAD2/3. *In vivo*, we found that HNK can suppress EMT in pancreatic cancer. HNK can also inhibit cancer cell migration along the nerve, reduce the damage to the sciatic nerve caused by tumor cells and protect the function of the sciatic nerve.

**Conclusion:**

Our results demonstrate that HNK can inhibit the invasion, migration, and PNI of pancreatic cancer by blocking SMAD2/3 phosphorylation, and we conclude that HNK may be a new strategy for suppressing PNI in pancreatic cancer.

## Introduction

Perineural invasion (PNI) is an important characteristic of pancreatic cancer (1). In the advanced stage of pancreatic cancer, the incidence of PNI is higher, and the incidence of PNI in pancreatic cancer has been reported to be 70%-100% (2). PNI can damage nerve structure and lead to cancer-associated pain (3). More importantly, PNI is considered a potential pathway for cancer cell dissemination and metastasis, similar to vascular and lymphatic channels (4). In the early stage of pancreatic cancer, the nerve density in the tumor area increases (5). As the tumor progresses, tumor cells surround and invade the nerve. Invasion of the nerves by cancer cells provides a pathway for the cancer cells to spread (4). PNI was identified as a relevant prognostic factor for both tumor recurrence after pancreatoduodenectomy and the survival outcome of pancreatic cancer patients (6, 7). A recent study showed that nerves can transport nutrients such as serine and glycine from areas with rich nutrients to areas with low nutrient levels, thereby promoting the growth of pancreatic cancer (8). Finding a therapeutic strategy that can inhibit PNI is a potential treatment direction for the treatment of pancreatic cancer.

SMAD2 and SMAD3 are two members of the SMAD family that mediate TGF-β signal transduction. TGF-β1 can activate downstream SMAD2/3 phosphorylation by binding to transmembrane serine-threonine kinase receptors on the cell membrane surface. Phosphorylated SMAD2/3 forms a complex that transfers into the nucleus, binds with DNA and regulates the transcription of target genes, thereby promoting tumor cell invasion, migration, and EMT (9). In our previous study, we found that metformin and lipoxin A4 can suppress the invasion ability and EMT in pancreatic cancer by blocking autocrine TGF-β1 signaling (10, 11).

Honokiol (HNK) is a polyphenolic compound that can be extracted from Magnolia species. HNK has multiple biological functions and possesses anti-inflammatory, antioxidative, antitumorigenic, and neuroprotective properties (12). Previous studies have indicated that HNK can suppress pancreatic cancer progression. HNK has been shown to suppress pancreatic cancer progression *via* miR-101 (13). In addition, HNK can interfere with the crosstalk between cancer cells and stromal cells, and this effect is achieved by inhibiting the expression of CXCR4 and SHH, thereby suppressing pancreatic cancer growth and metastasis (14). HNK can also inhibit EMT, migration, invasion, and angiogenesis in various cancers, including breast cancer (15), bladder cancer (16), lung cancer (17), and gastric cancer (18, 19). In addition, HNK can alleviate scale hyperplasia by inhibiting the phosphorylation of SMAD2/3 but does not inhibit the expression of TGF β (20). HNK has a significant analgesic activity by downregulating TRPV1 and P2Y receptors (21). HNK has anti-oxidative and anti-apoptotic effects in PC12 cells by regulating the GSK-3β and β-catenin signaling pathways (22). As HNK has both antitumor effects and neuroprotective functions, it might be an effective therapeutic strategy for PNI.

However, whether HNK has an inhibitory effect on PNI and its underlying mechanisms remain unknown. PNI is a continuous and multistep process (2). In this study, we focused on the three steps of PNI: (i) enhancement of the viability, mobility, and invasion of tumor cells; (ii) the crosstalk between cancer cells and nerves; (iii) cancer cells invading nerves and dissemination along nerves to investigate the effect of HNK on PNI and the potential mechanisms underlying this effect in pancreatic cancer.

## Materials and Methods

### Reagents

The antibodies used in this study were as follows: anti-N-cadherin (Proteintech), anti-E-cadherin (Proteintech), anti-Vimentin (Proteintech), anti-NGF (Abcam), anti-BDNF (Abcam), anti-p-SMAD2/3 (CST), anti-ACTIN (Proteintech), SMAD2 (CST), SMAD3(Beyotime), HRP-conjugated secondary antibodies (Proteintech), and fluorescence-conjugated secondary antibodies (Proteintech). Recombinant human TGF-β1 (10804-HNAC) and recombinant mouse TGF-β1 (80116-RNAH) were purchased from Sino Biological (Beijing, China). HNK was purchased from MedChem Express (Monmouth Junction, NJ, USA).

### Cell Lines

The human pancreatic cancer cell line PANC-1 was purchased from the Chinese Academy of Sciences Cell Bank of Type Culture Collection (Shanghai, China) and cultured in DMEM (Gibco, Grand Island, NY, USA) supplemented with 10% fetal bovine serum and 1% penicillin-streptomycin in an incubator with 5% CO2 at 37°C.

We used engineered transgenic *LSL-Kras* ^G12D/+^; *p53* ^fl/+^; *Pdx1-Cre* (KPC) mice, which harbor oncogenic *Kras* and *p53* inactivation specifically in the pancreas, and develop a spontaneous pancreatic cancer model. At the age of approximately three months, KPC mice developed pancreatic cancer. The pancreatic tumor was harvested from the KPC mouse, and the tumor tissue was separated into pieces with a diameter of approximately 1-2 mm with a scalpel. All the tissues were transferred to a centrifuge tube containing 5 mL washing solution for 1-2 minutes at room temperature, and the washing solution and the floating fatty tissue on the surface were discarded. Then, 5 mL of digestion solution was added to the centrifuge tube and incubated at 37°C for 4-6 hours. The supernatant was obtained, filtered with a 500-mesh filter, and centrifuged to obtain primary cells. The primary cells were then cultured in 1640 medium.

### MTT Assays

MTT (3-(4,5-dimethyl- 2-thiazolyl)-2,5-diphenyl-2-H-tetrazolium bromide) (Sigma, St. Louis, MO, USA) assays were used to measure the viability of pancreatic cancer cells. PANC-1 and KPC cells were seeded in 96-well plates at a density of 5,000 cells per well. All cells were incubated overnight in 10% FBS-containing medium at 37°C with 5% CO_2_. After treatment with HNK for 24 hours, 48 hours, and 72 hours, 20 μl of MTT solution (5 mg/ml in distilled water) was added to each well. The cells were then incubated for 4 hours, the medium was removed, and 200 μl of DMSO was added to each well. Then, the viability of the pancreatic cancer cells was assessed by measuring the optical density (OD) at 490 nm on a multifunctional microplate reader (POLARstar OPTIMA; BMG, Offenburg, Germany).

### Wound-Healing Assays and Transwell-Based Assays

A wound-healing assay was performed to examine the migration ability of pancreatic cancer cells. In brief, tumor cells were seeded into 6-well plates. After cancer cells reached an appropriate confluence, a wound was created with a 200-μL pipette tip. The cells were washed three times with PBS and then cultured with serum-free medium. Images of the wound were obtained at 0, 24 hours, and 48 hours. The healing area was used to evaluate the migration ability of tumor cells.

Fresh Matrigel was used to coat the upper face of the chamber, and 1×10^4^ cells suspended in serum-free medium were seeded in the upper wall of the chamber. Medium supplemented with 10% fetal bovine serum was added to the lower chamber. After culturing for 48 hours, the cells were fixed with 4% paraformaldehyde, and the cells that did not invade were removed with a cotton swab. The invading cells were stained with 1% crystal violet. Images were taken under a microscope, and the number of tumor cells in each field was recorded. Chambers not coated with Matrigel were used to assess the migration ability of the tumor cells.

### Cancer Cell-DRG Coculture Model

The cancer cell-DRG coculture model was used to assess nerve-cancer cell interactions. Newborn rats were purchased from the Laboratory Animal Center of Xi’an Jiaotong University. Alcohol (75%) was used for skin disinfection after euthanizing the newborn rat. The tissues on both sides of the spine were removed, and the spine was completely removed. The dorsal root ganglia were excised from the intervertebral foramen and placed into serum-free medium (DMEM/F12). The axons around the dorsal root ganglion and the capsule on the surface of the dorsal root ganglion were carefully removed under an anatomical microscope. The dorsal root ganglion was implanted into Matrigel, which was diluted 1:1 with DMEM/F12 medium. After the axons of the dorsal root ganglia grew, immunofluorescence (S100β, NF-200, TUJ1) was used to identify the dorsal root ganglia (**Supplementary Figure 1**).

After the identification of the dorsal root ganglion, we built a cancer cell-DRG coculture model. The previously described method was used to obtain dorsal root ganglia. Tumor cells were diluted 1×10^6^/mL after treatment, and 30 μL of the cell suspension was drawn and used to inoculate a 24-well plate. The dorsal root ganglion was inoculated at a distance of 2 mm from the edge of the cell drop. Then, Matrigel was added to cover the tumor cells and dorsal root ganglia so that a bridge was formed by the Matrigel between the tumor cells and dorsal root ganglia. The 24-well plate was placed in the incubator for 1 hour. After the Matrigel solidified, the medium supplemented with 10% FBS and 1% penicillin-streptomycin was carefully added, and half of the medium was replaced every two days. Images were taken every day to record the growth of the tumor cells and dorsal root ganglia. The ability of the tumor cells to migrate to the dorsal root ganglion was evaluated by the invasion index (α/γ), and the ability of the dorsal root ganglion axons to grow toward the tumor cells was evaluated by the growth index (β/γ), as shown in **Supplementary Figure 2**.

### Western Blotting

Cells and tumor tissue were lysed with RIPA buffer. The protein concentration was determined by a BCA protein assay kit. SDS-PAGE gels were used to electrophorese the total protein, which was then transferred to PDVF membranes (Roche, Penzberg, Germany). The membranes were blocked with 5% skimmed milk dissolved in TBST for 2 hours and incubated overnight at 4°C with the primary antibody. After washing three times with PBST, membranes were incubated with secondary antibody for 2 h at room temperature. The membranes were washed with TBST three times, and a ChemiDoc XRS System (Bio-Rad, CA, USA) was used to detect the expression of proteins with an enhanced chemiluminescence (ECL) kit (NCM Biotech, Suzhou, China).

### Immunofluorescence

Cells were washed with PBS and fixed with 4% paraformaldehyde. Triton X-100 (0.5%) was used to permeabilize the cells and 5% BSA was used for blocking. The cells were incubated with the primary antibody overnight at 4°C. The cells were then washed three times with TBST and incubated with fluorescence-conjugated secondary antibodies. After washing three times with PBST, the nuclei were stained with DAPI for 1 minute. Images were obtained with a Zeiss Instruments microscope.

### RNA Interference

Small interfering RNA targeting SMAD2 and SMAD3 (si-SMAD2 and si-SMAD3) to down-regulate the expression of SMAD2 and SMAD3 respectively were designed and synthesized by GenePharm (Shanghai, China). SMAD2-specific siRNAs (si-SMAD2-1#: sense: 5`-GGUGUUCGAUAGCAUAUUATT-3`, antisense: 5`-UAAUAUGCUAUCGAACACCTT-3`, and SMAD2-2#: sense:5`-CCCUGCAACAGUGUGUAAATT-3`, antisense 5`-UUUACACACUGUUGCAGGGTT-3`), SMAD3-specific siRNAs (si-SMAD3-1#: sense:5`-GCGUGAAUCCCUACCACUATT-3`, antisense 5`-UAGUGGUAGGGAUUCACGCTT-3`: and SMAD3-2#: antisense 5`-CGCAGGUUCUCCAAACCUATT-3`, antisense 5`-UAGGUUUGGAGAACCUGCGTT-3`), and negative control siRNA (sense: 5`-UUCUCCGAACGUGUCACGUTT-3`, antisense: 5`-ACGUGACACGUUCGGAGAATT-3`). Lipofectamine 2000 (Thermo Fisher Scientific, USA) was used to transfect siRNA into pancreatic cancer cells according to the manufacturer’s instructions.

### Quantitative Real-Time PCR

Total ribonucleic acid (RNA) was extracted with the Fastgen1000 RNA isolation system (Fastgen, Shanghai, China). Primescript RT reagent kit (TaKaRa, Dalian, China) was used to reverse transcribe the total RNA into cDNA. There are the PCR primer sequences: SMAD2, forward CGTCCATCTTGCCATTCACG, and reverse CTCAAGCTCATCTAATCGTCCTG; SMAD3 forward TGGACGCAGGTTCTCCAAAC and reverse CCGGCTCGCAGTAGGTAAC; NGF, forward TGTGGGTTGGGGATAAGACCA and reverse GCTGTCAACGGGATTTGGGT; BDNF, forward GGCTTGACATCATTGGCTGAC and reverse CATTGGGCCGAACTTTCTGGT; ACTIN, forward GCCGAGTGGAAACTTTTGTCG and reverse GGCAGCGTGTACTTATCCTTCT. The PCR amplification conditions: 10 min at 95°C followed by 40 cycles of at 95°C for 10 s, at 58°C for 10 s and 72°C for 20 s. We used β-ACTIN as the normalization control to detect the expression level of each target gene. The 2^-ΔΔCt^ method was used to calculate the relative gene expression.

### Subcutaneous Tumor Model and Perineural Invasion Model *In Vivo*

The KPC cell line was used to build the subcutaneous tumor model. KPC cells were resuspended in serum-free medium at 5×10^6^/mL, and 100 μL of cell suspension was injected subcutaneously into mice. When the tumors were macroscopic (approximately one week), the mice were divided into two groups (five mice in each group), the control group (saline and corn oil), and the HNK group (50 mg/kg/every day, diluted with corn oil). The size of the tumor was measured every week. After four weeks of drug administration, the mice were euthanized, tumor tissue was obtained, tumor tissue was weighed, and total protein was collected using RIPA buffer for use in the Western blot analysis.

A sciatic nerve invasion model was used to evaluate the interaction between tumor cells and nerves *in vivo* as described previously. Briefly, all mice were anesthetized, and their left sciatic nerve was exposed. KPC cells diluted to 1×10^7^/mL were injected into the sciatic nerve under a microscope with a Hamilton microsyringe (10 μL), and 2 μL was injected into each mouse. The model was successfully constructed in 8 mice, which were divided into two groups (four mice in each group). One week after cancer cell injection, HNK was injected every day as described for the subcutaneous tumor model. The function of the hind limbs of the mice was recorded every week. The extension length between the first and fifth toes of the hind limbs was calculated as the sciatic nerve function index. The distance of the tumor cells migrating along the sciatic nerve was measured from the injection point to the forefront of cancer cells. All protocols were approved by the Ethical Committee of the First Affiliated Hospital of Xi’an Jiaotong University, Xi’an, China.

### Statistical Analysis

Our data are shown as the mean ± SD. Student’s t-test *via* SPSS (version 15.0; SPSS, Chicago, IL, USA) was used to compare the difference between the two groups. *p*-values < 0.05 were considered significant.

## Results

### HNK Inhibits Pancreatic Cancer Malignant Behaviors and EMT

**Figure 1:**
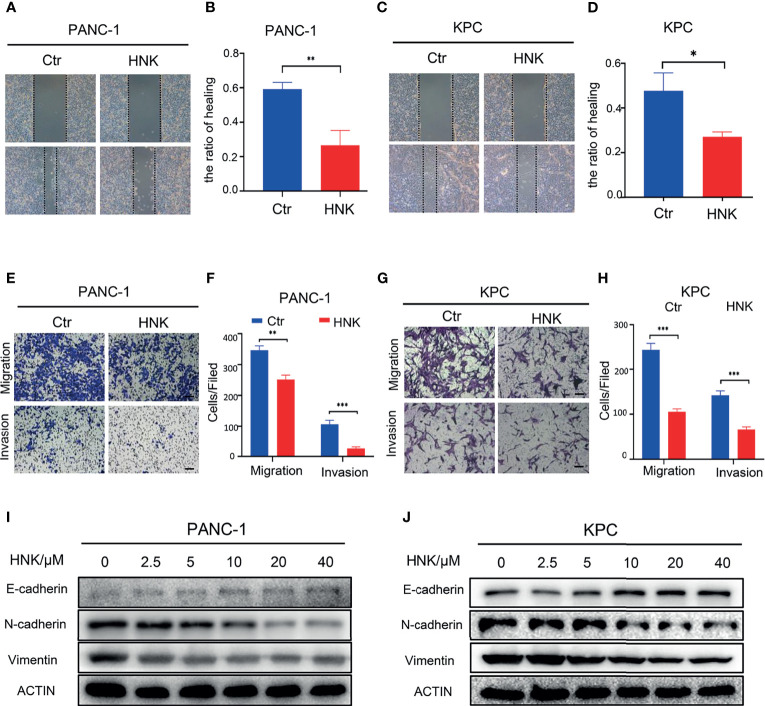
HNK inhibits pancreatic cancer malignant behaviors and EMT. **(A, B)** Wound healing assays of the control group and HNK group in PANC-1 cells, scale bar =200μm; **(C, D)** Wound healing assays of the control group and HNK group in KPC, scale bar = 200μm; **(E, F)** Migration assay and invasion assay of the control group and HNK group in PANC-1 cells, scale bar = 100μm; **(G, H)** Migration assay and invasion assay of the control group and HNK group in KPC, scale bar = 100μm; **(I, J)** Western blotting analysis of the expression of EMT-associated proteins (N-cadherin, E-cadherin, and Vimentin) in PANC-1 and KPC cells. *p < 0.05, **p < 0.01, ***p < 0.001.

In this study, we first investigated the effect of HNK on the migration and invasion ability of pancreatic cancer cells. The KPC cell line was used in this study. After the primary cells were passaged stably, we stained the KPC cells with immunofluorescent antibodies. The results showed that the KPC cells expressed both CK19 and amylase (**Supplementary Figure 3**). The KPC cell line was thus suitable for subsequent experiments. PANC-1 and KPC cell lines were treated with different concentrations of HNK (0, 2.5, 5, 10, 20, and 40 μM). MTT assays were used to assess the viability of pancreatic cancer lines at different time points (24 h, 48 h, 72 h, and 96 h). Our results showed that the viability of the pancreatic cancer cell line can be inhibited by HNK in a dose- and time-dependent manner (**Supplementary Figure 4**). A concentration of 10 μM was chosen for the wound healing assay and Transwell assay. The wound healing assays revealed that HNK inhibited the wound healing ability compared with that observed in the control group in PANC-1 (**Figures 1A, B**) and KPC (**Figures 1C, D**) cells. In other words, HNK can suppress the migration ability of pancreatic cancer cell lines. Transwell-based assays were performed to investigate the migration ability and invasion ability after HNK intervention. The migration and invasion results showed that HNK inhibited the migration and invasion capacity of PANC-1 (**Figures 1E, F**) and KPC cells (**Figures 1G, H**). In addition, different concentrations of HNK were used to treat pancreatic cancer lines, and the expression of EMT-associated markers was determined by Western blotting. Western blotting results showed that, in parallel with HNK concentration, the expression of N-cadherin and Vimentin was gradually downregulated, while HNK promoted the expression of E-cadherin in PANC-1 (**Figure 1I**) and KPC (**Figure 1J**) cells. Collectively, these results indicated that HNK can inhibit pancreatic cancer malignant behaviors and EMT.

### HNK Suppresses PNI and SMAD2/3 Signaling in Pancreatic Cancer

**Figure 2:**
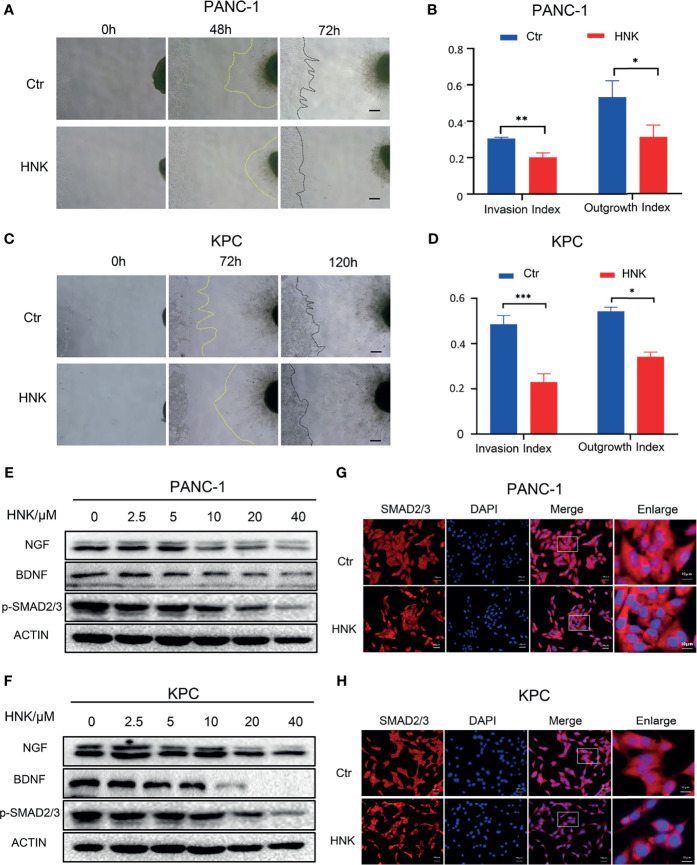
HNK suppresses perineural invasion and SMAD2/3 signaling in pancreatic cancer. **(A, B)** Cancer cell-DRG coculture model of the control group and HNK group in PANC-1 cells, scale bar = 200μm; **(C, D)** Cancer cell-DRG coculture model of the control group and HNK group in KPC cells, scale bar = 200μm; **(E)** Western blotting analysis of the expression of NGF, BDNF, and p-SMAD2/3 in PANC-1 cells; **(F)** Western blotting analysis of the expression of NGF, BDNF, and p-SMAD2/3 in KPC cells; **(G, H)** Immunofluorescence staining SMAD2/3 on PANC-1 and KPC cell lines. *p < 0.05, **p < 0.01, ***p < 0.001.

The interaction between nerve cells and tumor cells also plays an important role in the process of PNI. To further investigate the effect of HNK on PNI, a cancer cell-DRG coculture model was used to assess the interaction between cancer cells and nerves *in vitro*. The live-cell imaging system showed that the KPC cell line can migrate to the dorsal root ganglion, and the dorsal root ganglion axons can also grow toward tumor cells (**Supplementary Video 1**). In addition, fluorescence microscopy showed that KPC cells migrated along the axon after coming into contact with the axon of the dorsal root ganglion (**Supplementary Figure 5**). Overall, these results indicate that KPC cells can be used for PNI research. The results of the cancer cell-DRG coculture system showed that HNK can inhibit cancer cell migration toward the DRG and that the DRG axon length was significantly lower in the group treated with HNK. HNK inhibited the invasion index and the growth index compared to that observed in the control cells, and the differences were statistically significant in PANC-1 (**Figures 2A, B**) and KPC (**Figures 2C, D**) cells. In addition, neurotrophic factors were related to PNI. After pancreatic cancer lines were treated with different concentrations of HNK, Western blotting was applied to detect the expression of NGF and BDNF. We found that HNK inhibited the expression of NGF and BDNF in PANC-1 (**Figure 2E**) and KPC cells (**Figure 2F**). We further investigated whether HNK treatment has an impact on the activity of SMAD2/3 signaling. The results showed that the levels of p-SMAD2/3 in PANC-1 and KPC cells were downregulated after HNK treatment in PANC-1 (**Figure 2E**) and KPC (**Figure 2F**) cells. Then, immunofluorescence was used to detect the effect of HNK on SMAD2/3 signaling. We found that HNK prevented the translocation of SMAD2/3 to the nucleus (**Figures 2G, H**). These data suggested that HNK treatment inhibits PNI and SMAD2/3 signaling in pancreatic cancer cells.

### Activating the SMAD2/3 Signaling Can Promote PNI in Pancreatic Cancer

**Figure 3:**
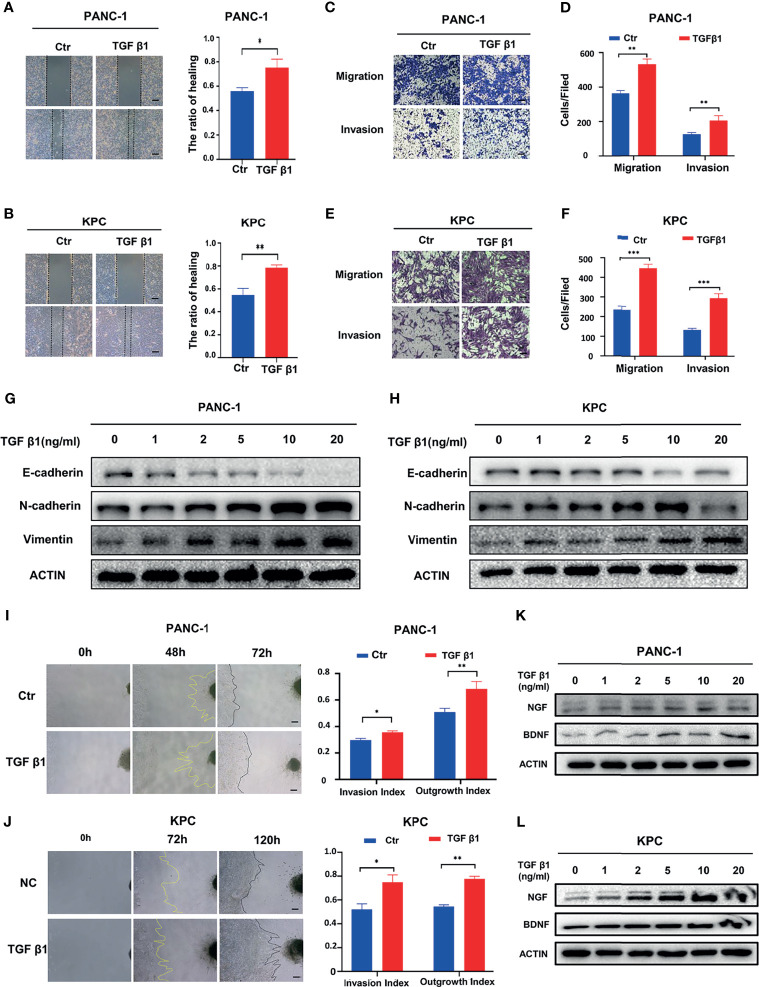
Activating SMAD2/3 signaling can promote perineural invasion. **(A, B)** Wound healing assays of the control group and TGF β1 group, scale bar = 200μm; **(C-F)** Migration assay and invasion assay of the control group and TGF β1 group, scale bar = 100μm; **(G, H)** Western blotting analysis of the expression of N-cadherin, E-cadherin, Vimentin; **(I, J)** Cancer cell-DRG coculture model of the control group and TGF β1 group, scale bar = 200μm; **(K, L)** Western blotting analysis of the expression of NGF and BDNF. *p < 0.05, **p < 0.01, ***p < 0.001.

To understand the effect of SMAD2/3 signaling in PNI, we further investigated whether activating SMAD2/3 signaling has an impact on PNI. Recombinant TGF-β1 (10 ng/mL) was used to treat PANC-1 and KPC cells to activate SMAD2/3 signaling. Wound healing assays showed that the activation of SMAD2/3 can promote the wound healing ability of PANC-1 (**Figure 3A**) and KPC (**Figure 3B**) cells. The results of the Transwell-based assays showed that the migration and invasion capabilities of cancer cells were increased by recombinant TGF-β1 treatment (**Figures 3C–F**). Western blotting results showed that TGF-β1 upregulated the expression of N-cadherin and Vimentin and downregulated the expression of E-cadherin in PANC-1 (**Figure 1G**) and KPC cells (**Figure 1H**). Further investigation of the effect of SMAD2/3 activation on PNI in pancreatic cancer revealed that activating SMAD2/3 signaling increased the cancer cell invasion index and the DRG axon growth index (**Figures 3I, J**). In addition, Western blotting results showed that activating SMAD2/3 can promote the protein expression of NGF and BDNF (**Figures 3K, L**). We hypothesize that activating SMAD2/3 can promote PNI in pancreatic cancer.

**Figure 4:**
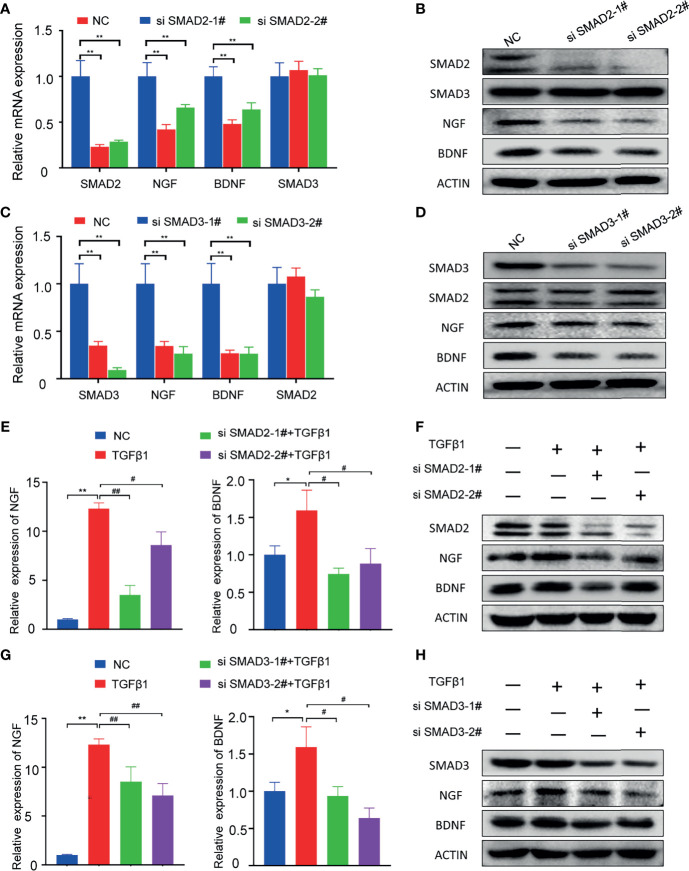
Knockdown of SMAD2 or SMAD3 can inhibit the expression of NGF and BDNF. **(A, B)** qPCR and Western blotting analysis of the expression of SMAD2, SMAD3, NGF, BDNF in PANC-1 cell line after treated with SMAD2-specific siRNAs; **(C, D)** qPCR and Western blotting analysis of the expression of SMAD3, SMAD2, NGF, BDNF in PANC-1 cell line after treated with SMAD3-specific siRNAs; **(E)** qPCR analysis of the expression of NGF, BDNF in PANC-1 cell line after treated with TGFβ-1 and SMAD2-specific siRNAs; **(F)** Western blotting analysis of the expression of SMAD2, NGF, BDNF in PANC-1 cell line after treated with TGFβ-1 and SMAD2-specific siRNAs; **(G)** qPCR analysis of the expression of NGF, BDNF in PANC-1 cell line after treated with TGFβ-1 and SMAD3-specific siRNAs; **(H)** Western blotting analysis of the expression of SMAD3, NGF, BDNF in PANC-1 cell line after treated with TGFβ-1 and SMAD3-specific siRNAs. *p < 0.05, **p < 0.01; ^#^ *p* < 0.05, ^##^ *p* < 0.01.

To explore whether SMAD2 or SMAD3 is responsible for the PNI of pancreatic cancer, we designed siRNA to knockdown SMAD2 and SAMD3 in PANC-1 cell line respectively. The qPCR results showed that SMAD2-specific siRNAs can knock down the mRNA expression of SMAD2 but not SMAD3, and the mRNA expression levels of NGF and BDNF decreased when SMAD2 is knocked down (**Figure 4A**). Western blotting results showed that SMAD2-specific siRNAs can knock down SMAD2 protein expression specifically and the protein expression of NGF and BDNF were decreased (**Figure 4B**). SMAD3-specific siRNAs can knock down SMAD3 expression specifically and also can knock down the expression of NGF and BDNF (**Figures 4C, D**). In addition, we further investigated whether knocking down the expression of SMAD2 and SMAD3 can block the expression of neurotrophic factors promoted by TGF-β1. We found that knockdown of either SMAD2 or SMAD3 can lead to the inhibition of NGF and BDNF expression compared with the TGF-β1 treated cells (**Figures 4E–H**). These results suggested that both SMAD2 and SMAD3 play important roles in regulating the expression of NGF and BDNF.

### HNK Suppresses PNI by Inhibiting SMAD2/3 Signaling

**Figure 5:**
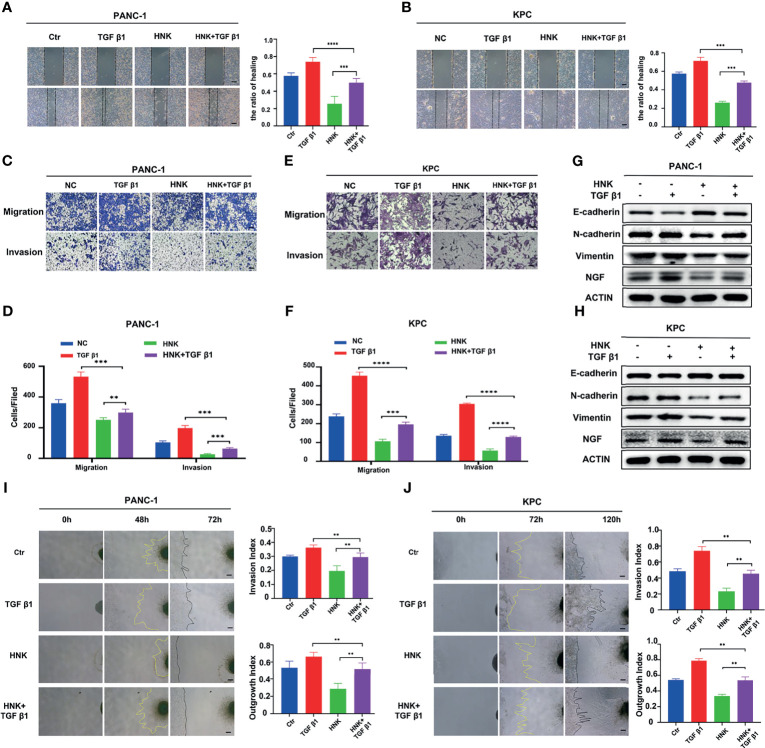
HNK suppresses perineural invasion by inhibiting SMAD2/3 signaling. **(A, B)** Wound healing assays of the control group, HNK group, TGF β1 group, and HNK+TGF β1 group, scale bar = 200 μm; **(C–F)** Migration assay and invasion assay of the control group, HNK group, TGF β1 group, and HNK+TGF β1 group, scale bar = 100 μm; **(G, H)** Western blotting analysis of the expression of NGF, N-cadherin, E-cadherin, and Vimentin. **(I, J)** Cancer cell-DRG coculture model of the control group, HNK group, TGF β1 group, and HNK+TGF β1 group, scale bar = 200 μm; **p < 0.01, ***p < 0.001, ****p < 0.0001.

To investigate whether HNK suppresses PNI mediated by inhibiting SMAD2/3 signaling, PANC-1 and KPC cells were treated with HNK and recombinant TGF-β1. Wound healing results showed that activating SMAD2/3 with TGF-β1 recovered the wound healing ability that was suppressed by HNK treatment in PANC-1 (**Figure 5A**) and KPC (**Figure 5B**) cells. The results of the Transwell-based assays also showed that activating SMAD2/3 could recover the migration and invasion capacity inhibited by HNK in PANC-1 (**Figures 5C, D**) and KPC (**Figures 5E, F**) cells. In addition, activating SMAD2/3 restored the expression of N-cadherin, Vimentin, and NGF, which were suppressed by HNK, and inhibited the expression of E-cadherin, which was increased by HNK in PANC-1 (**Figure 5G**) and KPC cells (**Figure 5H**). In the cancer cell-DRG coculture system, we found that the inhibitory effect of HNK on the invasion index and the growth index was abolished by recombinant TGF-β1 in PANC-1 cells (**Figure 5I**) and KPC cells (**Figure 5J**). These results showed that activating SMAD2/3 can reverse the effect of HNK. Therefore, we revealed that HNK suppresses perineural invasion by inhibiting SMAD2/3 signaling in pancreatic cancer.

### HNK Inhibits PNI in Pancreatic Cancer *In Vivo*

**Figure 6:**
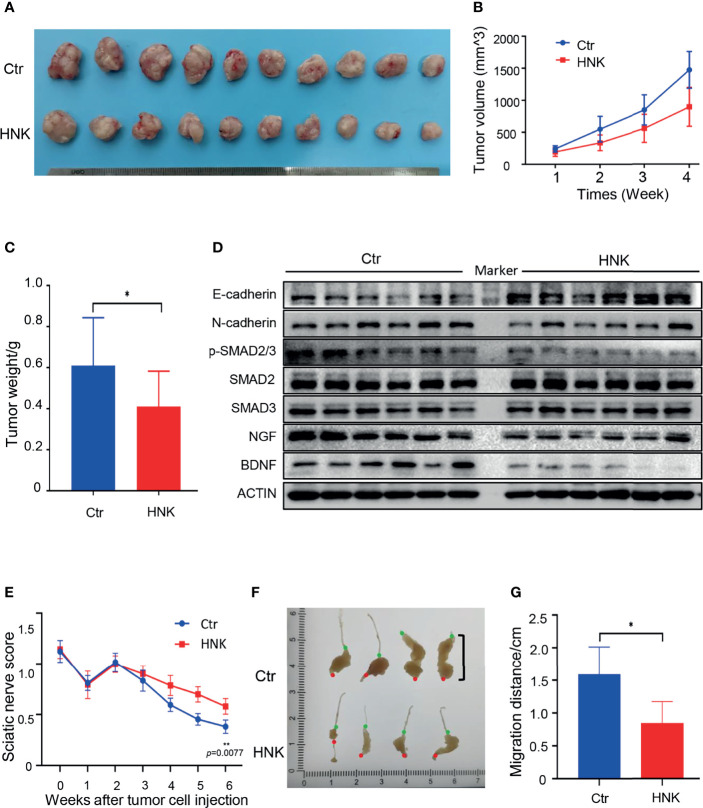
HNK inhibits EMT and perineural invasion *in vivo*. **(A)** Photographs of tumors in the subcutaneous pancreatic cancer model. **(B)** Tumor volumes were measured every week. **(C)** Tumor weights after sacrificing mice in the subcutaneous pancreatic cancer model. **(D)** Western blotting analysis of the expression of E-cadherin, N-cadherin, NGF, BDNF, and the activity of p-SMAD2/3 in tumor tissues. **(E)** The sciatic nerve function index in the sciatic nerve invasion model. **(F)** Photographs of tumors in the sciatic nerve (red dot: the injection point; green dot: the forefront of tumor cells). **(G)** Migration distances of tumors along the sciatic nerve were measured (from the injection point to the forefront of tumor cells). *p < 0.05, **p < 0.01.

HNK can significantly inhibit the invasion, migration, and PNI of pancreatic cancer *in vitro*. The therapeutic effect of HNK was demonstrated *in vivo.* The results of the subcutaneous pancreatic cancer model showed that tumor growth induction was successful and that the weights of the tumors were reduced when the mice were treated with HNK (**Figures 6A, C**). In addition, we observed and measured the volume of the subcutaneous tumors every week, and the results showed that HNK can significantly inhibit the tumor volume (**Figure 6B**). Western blotting results indicated that HNK can increase the expression of E-cadherin and inhibit the expression of N-cadherin, NGF, BDNF, and the activity of p-SMAD2/3 *in vivo* (**Figure 6D**). We next investigated the effect of HNK on the process of PNI *in vivo.* We established a sciatic nerve invasion model by injecting pancreatic cancer cells into the sciatic nerve. The sciatic nerve function index was measured every week. The results showed that the sciatic nerve function index of the HNK group was higher than that of the control group (**Figure 6E**). In other words, HNK can protect the function of the hind limbs. Six weeks after cancer cell injection, mice were euthanized to obtain the sciatic nerve, and we found that tumor cells migrated along the sciatic nerve (**Figure 6F**). By measuring the distance of the tumor cells migrating along the sciatic nerve which was from the injection point to the forefront of tumor cells, we determined that HNK can significantly inhibit cancer cell migration along the sciatic nerve (**Figure 6G**). The above results indicate that HNK can inhibit EMT and PNI in pancreatic cancer *in vivo.*

## Discussion

Pancreatic cancer has a dismal prognosis, and invasion and distant metastasis is a major cause of cancer-associated death worldwide. Pancreatic cancer is typically difficult to treat, and few effective treatment strategies are available, because of the lack of effective early diagnostic measures. In addition, approximately 50% of patients present with distant metastases at the time of diagnosis (23). Surgical resection provides the only chance of cure, reaching a 5-year survival rate of 25% (24). However, pancreatic cancer has a high frequency of recurrence even after curative resection. PNI is considered to be a distant metastasis (4) and a reason for the recurrence (6) of pancreatic cancer. Cancer cells invade and spread along the nerves, which may be a mechanism of distant metastasis of pancreatic cancer; cancer cells remaining in the nerves of patients undergoing curative surgery may be an important reason for the high-frequency recurrence rate of pancreatic cancer (25). Inhibition of PNI may be a strategy to inhibit distant metastasis and reduce the recurrence rate of pancreatic cancer.

PNI is a continuous and multistep process. The enhancement of the viability, mobility, and invasion capacity of tumor cells is an important process in PNI. The high invasion and migration characteristics of PDAC contribute to PNI (2). Inhibiting the migration and invasion ability of pancreatic cancer cells may be a strategy to inhibit PNI. In this study, we found that HNK can inhibit the migration and invasion capacity of pancreatic cancer cells. In addition, EMT is associated with migration and invasion, and suppressing EMT can inhibit the migration and invasion capacities of pancreatic cancer. In our study, we found that HNK can downregulate the expression of mesenchymal markers (N-cadherin and vimentin) but upregulate the expression of an epithelial marker (E-cadherin) *in vitro*. These results demonstrated that HNK might attenuate PNI by inhibiting the invasion, migration, and EMT of pancreatic cancer cells.

The crosstalk between nerve cells and tumor cells also plays an important role in the process of PNI (2). A variety of neurotrophic factors are involved in the crosstalk between nerves and pancreatic cancer cells. Studies have shown that pancreatic cancer cells can secrete neurotrophic factors, including nerve growth factor (NGF), brain-derived neurotrophic factor (BDNF), and glial cell line-derived neurotrophic factor (GDNF), to promote neuroplasticity and thereby recruit nerves and facilitate tumor cell invasion of nerves. A previous study revealed that in genetically engineered mice harboring spontaneous pancreatic cancer, *Ngf* overexpression led to prominent PNI and enlarged intratumoral nerves. In addition, *Ngf* overexpression accelerated tumor formation and reduced the median overall survival time (26). Our previous studies revealed that the activation of the HGF/c-Met pathway can upregulate the expression of NGF and facilitate perineural invasion (27). In addition, LOXO-101 (a Trk-NGF inhibitor) can reduce innervation and decrease PDAC tumor burden in an orthotopic model (8). Aditi A. et al. also proved that blocking NGF can inhibit pancreatic cancer PNI *in vitro* (28). The results of our study showed that HNK can inhibit the expression of NGF and BDNF. In the cancer cell-DRG coculture system, we found that HNK can suppress the crosstalk between nerve cells and tumor cells, thereby inhibiting PNI in pancreatic cancer.

Pancreatic cancer cells invading into nerves and migrating along nerves are important processes of PNI (25). HNK can exert its antitumorigenic effects in the central nervous system because it can cross the blood-brain barrier to increase its bioavailability in neurological tissues (29). Interestingly, HNK has been shown to exhibit minimal cytotoxicity against normal cell lines (30, 31). As HNK has both antitumor effects and bioavailability in neurological tissues, it might be an effective therapeutic strategy for PNI. In our study, using the sciatic nerve invasion model, we found that HNK can inhibit cancer cell migration along the sciatic nerve. In addition, HNK can reduce the damage caused by tumor cells to the sciatic nerve and protect the function of the sciatic nerve after invasion by tumor cells. The results indicated that HNK has a good anti-cancer effect even when pancreatic cancer cells have invaded the nerves. However, the mechanism by which HNK reduces the damage caused by tumor cells to the sciatic nerve needs further study.

It is well known that elevated TGF-β signaling promotes cancer metastasis and EMT. Canonical TGF-β signaling requires the activation of SMAD2 and SMAD3 by TGF-β. Once phosphorylated, the SMAD complex translocates into the nucleus, thus regulating the transcription of target genes, including EMT-associated genes. SMAD2/3 activation by TGF-β can induce the transcription of snail protein and increasing the EMT (15). Here, we report that activating SMAD2/3 by TGF-β1 can also induce EMT and the invasion and migration of pancreatic cancer cells. More importantly, activating SMAD2/3 can upregulate the expression of NGF and BDNF in pancreatic cancer cells and promote the PNI of pancreatic cancer *in vitro*. In the cancer cell-DRG coculture system, we found that activating SMAD2/3 of pancreatic cancer cells can promote the crosstalk between cancer cells and nerves by increasing the cancer cell invasion index and the DRG axon growth index. TGF-β1/SMAD2/3 signaling contributes to PNI in pancreatic cancer. However, it is unclear whether SAMD2 or SMAD3 is responsible for the PNI of pancreatic cancer. In our study, we found that knockdown of either SMAD2 or SMAD3 can lead to the inhibition of NGF and BDNF expression. SAMD2 and SMAD3 are both responsible for the PNI of pancreatic cancer. In addition, we also found that knockdown of either SMAD2 or SMAD3 can partially inhibit the expression of NGF and BDNF promoted by TGF-β1. These results suggested that TGF-β1 may promote the PNI of pancreatic cancer through both SMAD-independent and SMAD-dependent pathways. In the next study, we will focus on the mechanism of TGF-β1 and SMAD2/3 regulating the PNI of pancreatic cancer and the underlying mechanisms need further exploration.

The anticancer activity of HNK is mediated by various mechanisms in various tumors. HNK can inhibit breast cancer cell metastasis by blocking EMT through downregulating Snail/Slug protein translation (15). Honokiol inhibits the migration of renal cell carcinoma through activation of the RhoA/ROCK/MLC signaling pathway (32) and by targeting KISS1/KISS1R signaling (33). Honokiol can induce apoptosis and autophagy *via* the ROS/ERK1/2 signaling pathway in human osteosarcoma cells (34). In addition, the functions of HNK on EMT and the regulation of SMAD2/3 have been reported in multiple papers. The results of Lv et al. showed that HNK treatment can decrease the SMAD2/3 phosphorylation and HNK can inhibit EMT-mediated motility and migration of human non-small cell lung cancer cells *in vitro (* 17). HNK also can ameliorate pulmonary fibrosis by inhibiting TGF-β/SMAD signaling (35). In our study, we found that HNK can inhibit the PNI of pancreatic cancer in several aspects *via* regulating SMAD2/3 signaling. On the one hand, HNK can inhibit the viability, mobility, and invasion of tumor cells *via* inhibiting the SMAD2/3 signaling. On the another hand, HNK can block the crosstalk between cancer cells and nerves by suppressing the activating of SMAD2/3 signaling. However, although SMAD2/3 phosphorylation is changed during HNK treatment, the molecular mechanism is still never mentioned. The prediction of molecular docking models and the development of related experiments may further clarify the mechanism. According to the structure and function of SMAD2/3, focus on the mechanisms whether HNK and SMAD2/3 can form intermolecular hydrogen bonds and the phosphorylation of SMAD2/3 is inhibited are important.

Although the mechanism of HNK inhibiting PNI of pancreatic cancer is revealed by these studies, there are also limitations. In our study, the subcutaneous cancer model cannot precisely recapitulate the microenvironment of pancreatic cancer growth *in vivo* and cannot mimic aspects of tumor invasion into the nerves or metastasis to other target organs *in vivo*. Therefore, a more appropriate invasion model needs to exploring to investigate mechanisms of the PNI *in vivo*. In addition, siRNA can only get partial knockdown but not knockout SMAD2 and SMAD3. Maybe SMAD2^-/-^ and SMAD3^-/-^ cells can help us understand more clearly the mechanism by which whether TGF-β1 promotes the expression of NGF and BDNF through both SMAD-independent and SMAD-dependent pathways. Whether SMAD2/3 can promote the transcription of NGF and BDNF by directly binding to NGF and BDNF promoters also need to be explored.

In conclusion, our results indicated that HNK can suppress PNI of pancreatic cancer by affecting three important steps of PNI: (i) inhibiting the EMT, invasion, and migration of pancreatic cancer cells; (ii) blocking the crosstalk between cancer cells and nerves; (iii) inhibiting cancer cells disseminating along nerves. HNK has the potential to inhibit the PNI of pancreatic cancer *in vitro* and *in vivo*. HNK has good bioavailability in neurological tissues and has neuroprotective effects, and may inhibit distant metastasis and postoperative recurrence of pancreatic cancer *via* suppression of PNI. In addition, our results show that HNK suppresses perineural invasion of pancreatic cancer by inhibiting SMAD2/3 signaling. We conclude that HNK may be a new strategy for suppressing PNI in pancreatic cancer.

## Data Availability Statement

The original contributions presented in the study are included in the article/**Supplementary Material**. Further inquiries can be directed to the corresponding authors.

## Ethics Statement

The animal study was reviewed and approved by The Ethical Committee of the First Affiliated Hospital of Xi’an Jiaotong University.

## Author Contributions

Conception and design: TQ, ZW, HS, QM, and LH. Development of methodology: TQ, JL, YX, XW, MG, QW, ZZ, SZ, WZ, and FC. Acquisition and analysis of data: TQ, YX, JL, and XW. Writing, review, and or revision of the manuscript: TQ, YX, HS, and QM. Administrative, technical, or material support: QM, ZW, FC, and LH. Study supervision: QM, ZW, FC, and LH. All authors contributed to the article and approved the submitted version.

## Funding

This work was supported by grants from the National Natural Science Foundation of China (No.82072699); Natural Science Basic Research Program of Shaanxi (No. 2020JQ-510); Science and Technology Project of Xi`an [No. 20YXYJ0002 (8)].

## Conflict of Interest

The authors declare that the research was conducted in the absence of any commercial or financial relationships that could be construed as a potential conflict of interest.

## Publisher’s Note

All claims expressed in this article are solely those of the authors and do not necessarily represent those of their affiliated organizations, or those of the publisher, the editors and the reviewers. Any product that may be evaluated in this article, or claim that may be made by its manufacturer, is not guaranteed or endorsed by the publisher.

## Supplementary Material

Supplementary Figure 1 After the axons of the dorsal root ganglia grow, immunofluorescence staining the markers of S100β, NF-200, TUJ1.

Supplementary Figure 2 The ability of tumor cells migrating to the dorsal root ganglion is evaluated by the invasion index (α/γ), and the ability of dorsal root ganglion axons to grow toward tumor cells is evaluated by growth index(β/γ).

Supplementary Figure 3 Immunofluorescence was used to identify the KPC cell line *via* staining the markers of CK19 and Amylase.

Supplementary Figure 4 KPC and PANC-1cell lines were treated with different concentrations of HNK (0,2.5, 5,10,20,40μM). MTT assays were used to assess the viability of pancreatic cancer lines at different time points (24h,48h,72h, and 96h).

Supplementary Figure 5 Fluorescence microscopy showed that KPC cells can migrate along the axon after contacting the axon of the dorsal root ganglion.

Supplementary Video 1 The live-cell imaging system was used to record the KPC cell line and the dorsal root ganglion.
