# Supplementary figures and images for: Honokiol Suppresses Perineural Invasion of Pancreatic Cancer by Inhibiting SMAD2/3 Signaling
Source: Front Oncol. 2021 Oct 4;11:728583. doi: 10.3389/fonc.2021.728583 (PMC8521150; doi:10.3389/fonc.2021.728583)

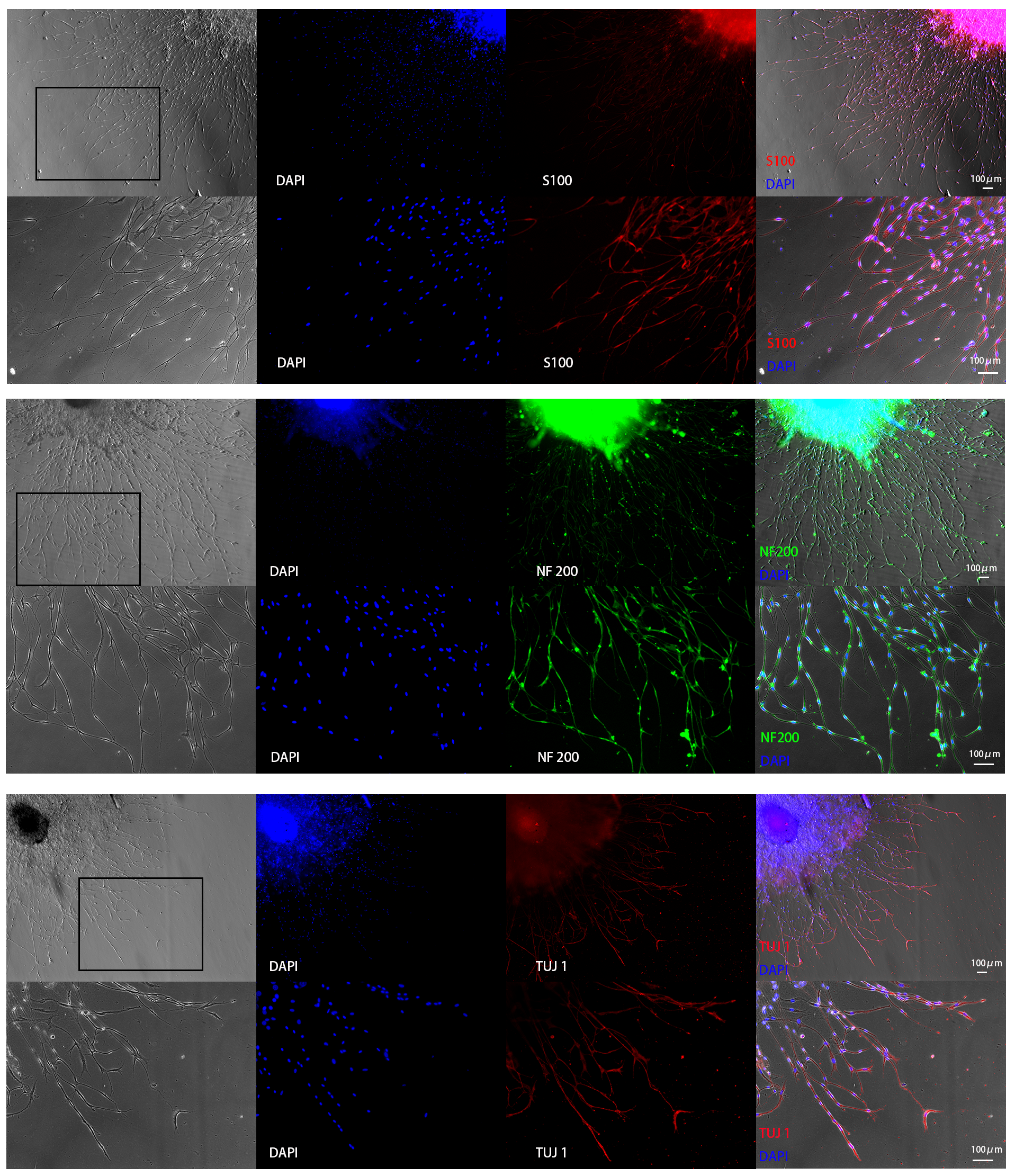

Supplement: Supplementary Figure 1 — After the axons of the dorsal root ganglia grow, immunofluorescence staining the markers of S100β, NF-200, TUJ1. [file Image_1.tif]

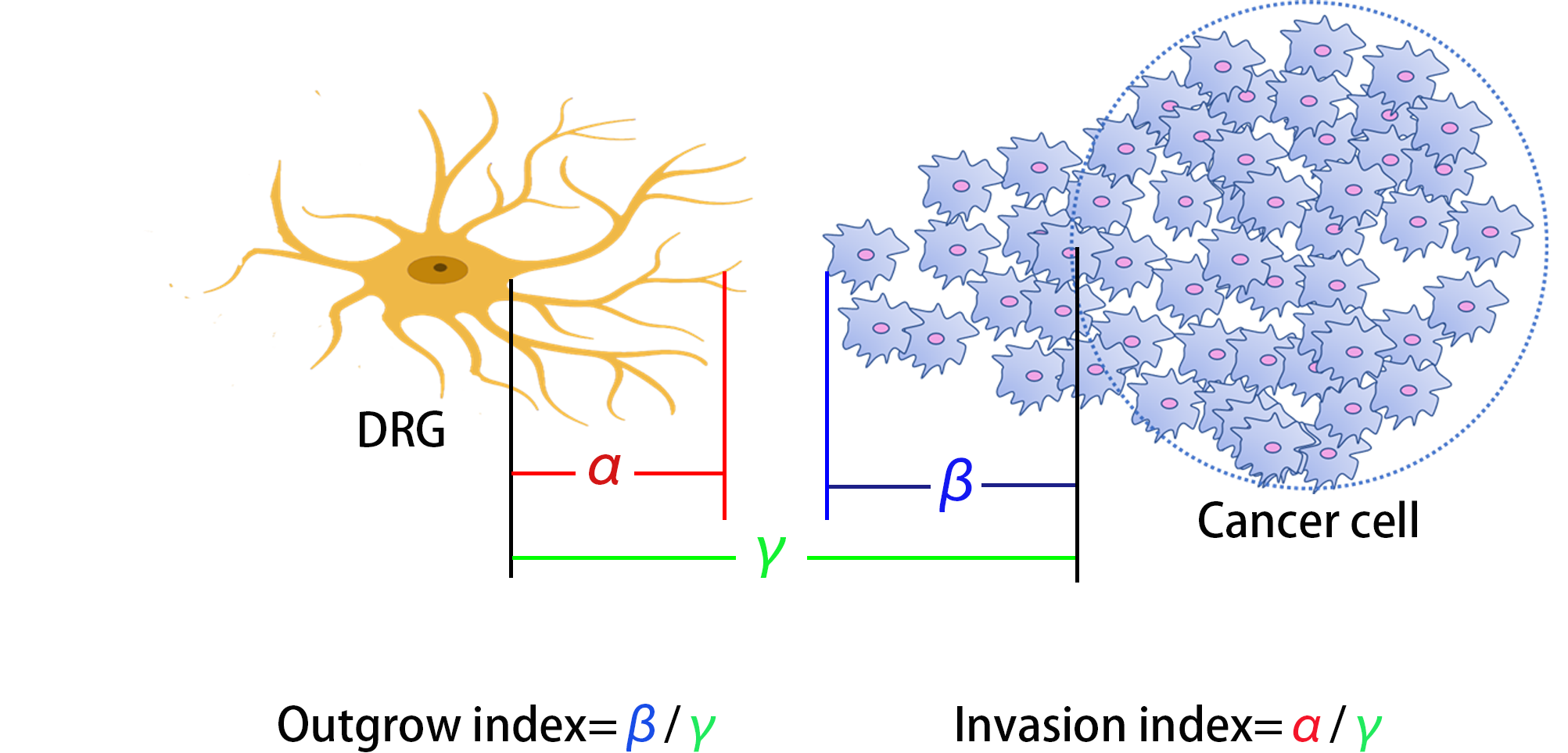

Supplement: Supplementary Figure 2 — The ability of tumor cells migrating to the dorsal root ganglion is evaluated by the invasion index (α/γ), and the ability of dorsal root ganglion axons to grow toward tumor cells is evaluated by growth index(β/γ). [file Image_2.tif]

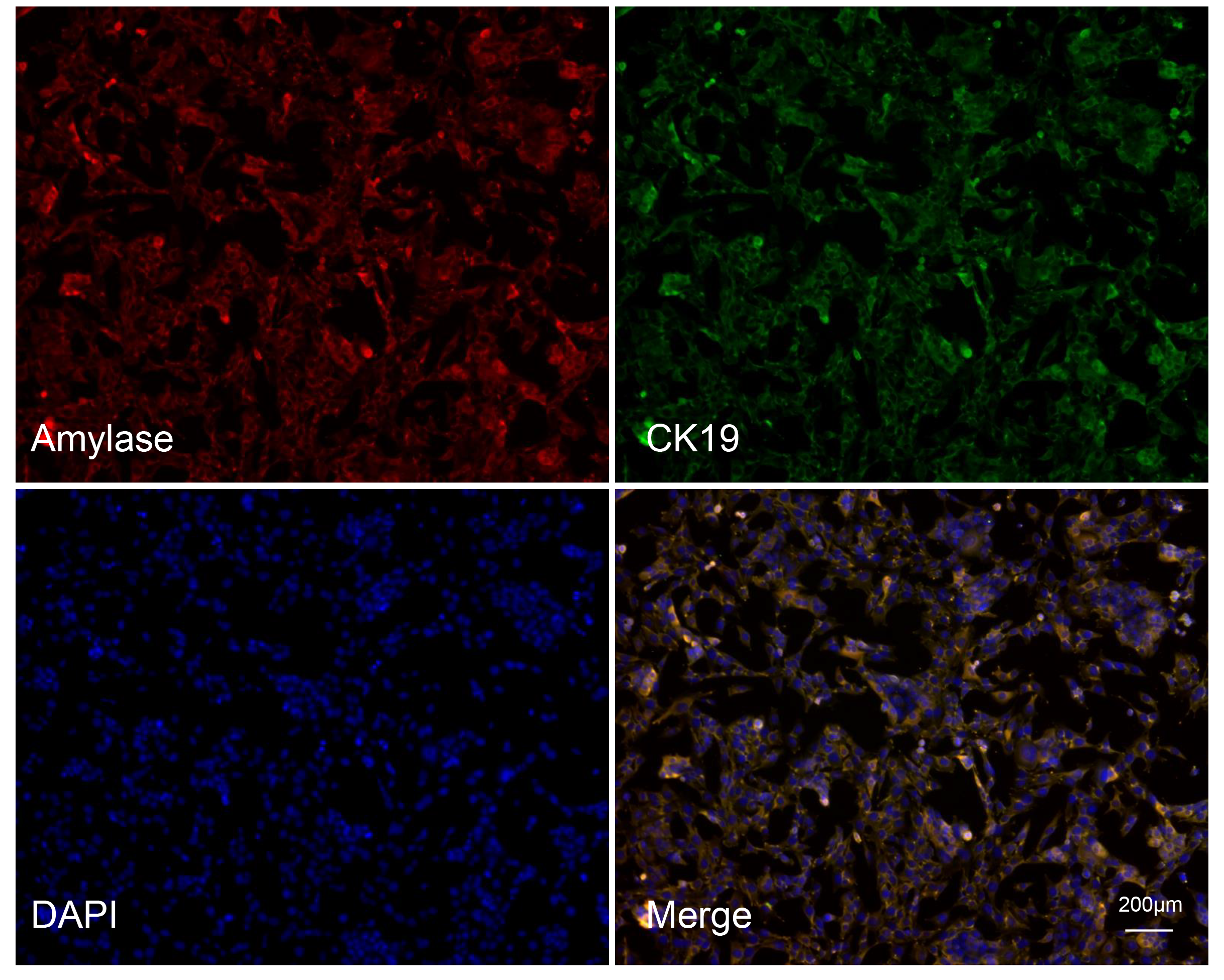

Supplement: Supplementary Figure 3 — Immunofluorescence was used to identify the KPC cell line via staining the markers of CK19 and Amylase. [file Image_3.tif]

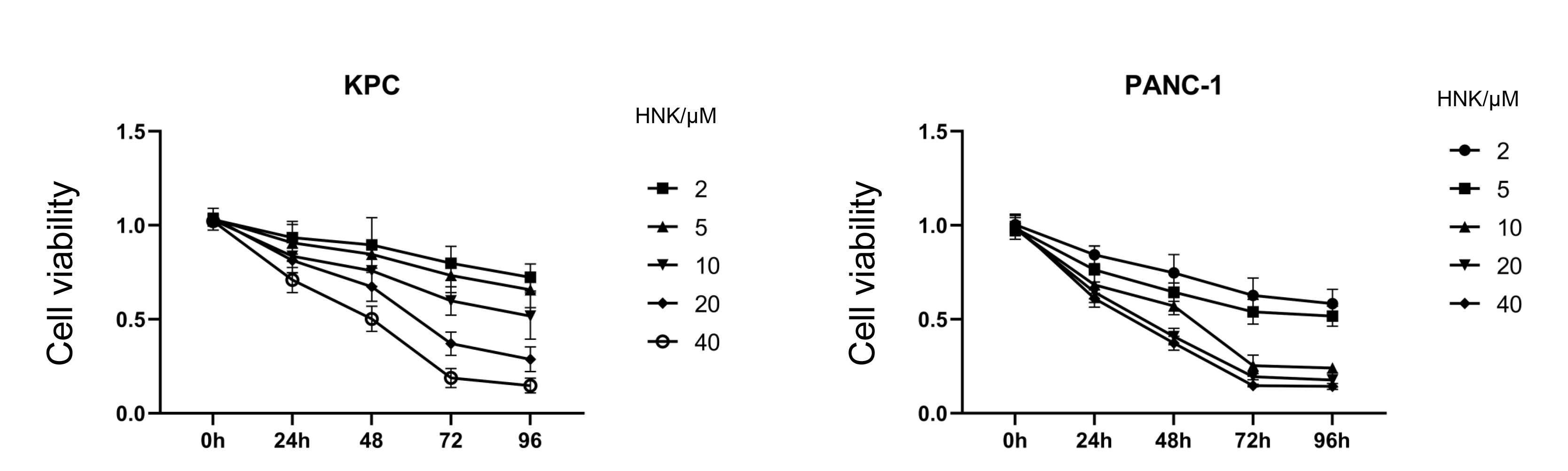

Supplement: Supplementary Figure 4 — KPC and PANC-1cell lines were treated with different concentrations of HNK (0,2.5, 5,10,20,40μM). MTT assays were used to assess the viability of pancreatic cancer lines at different time points (24h,48h,72h, and 96h). [file Image_4.tif]

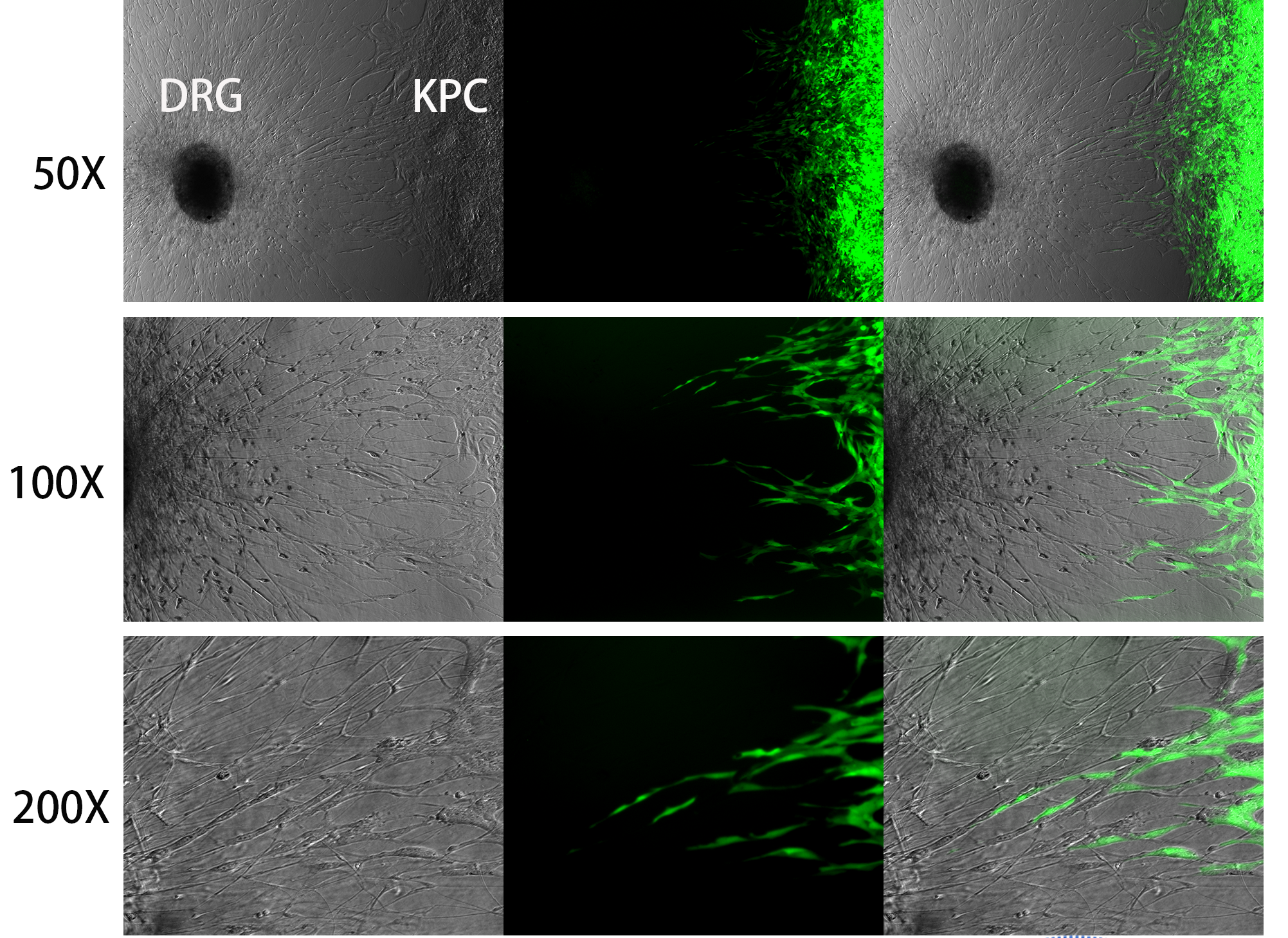

Supplement: Supplementary Figure 5 — Fluorescence microscopy showed that KPC cells can migrate along the axon after contacting the axon of the dorsal root ganglion. [file Image_5.tif]

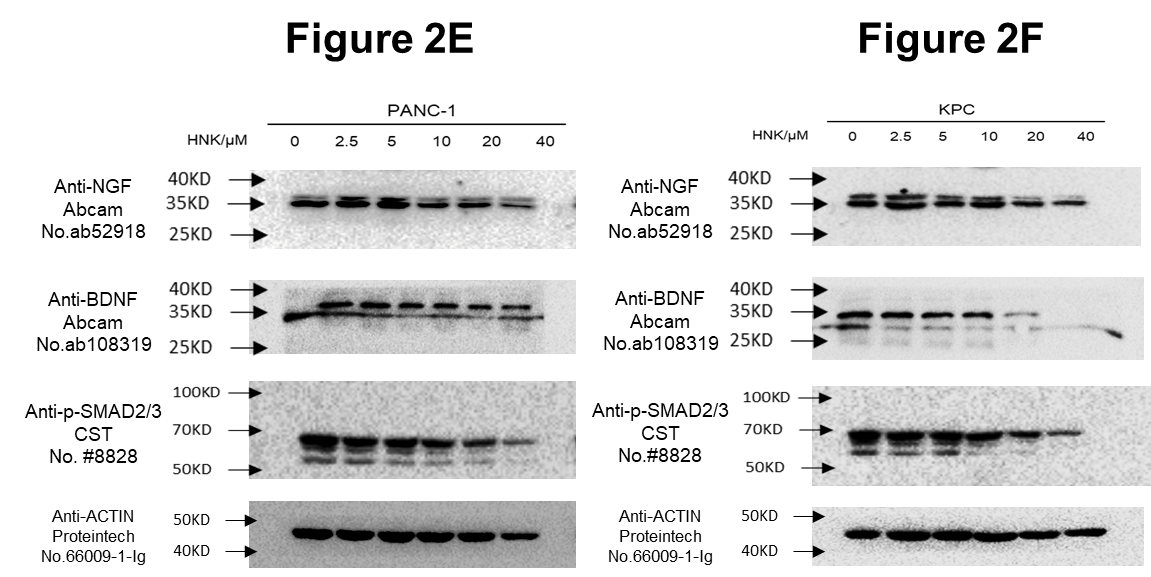

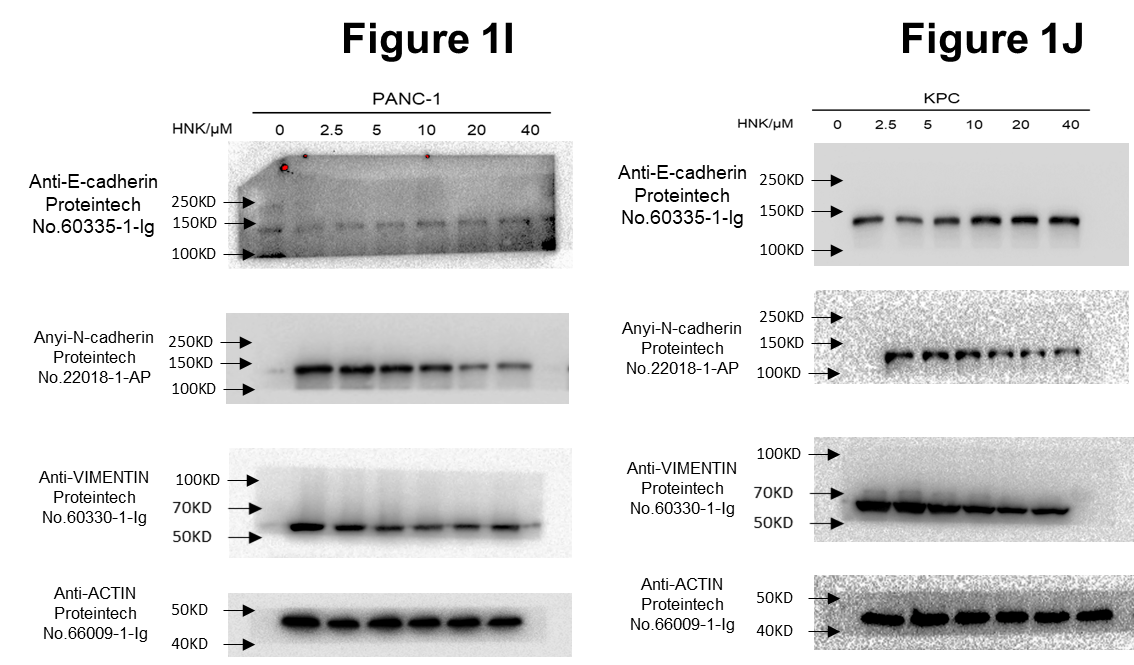


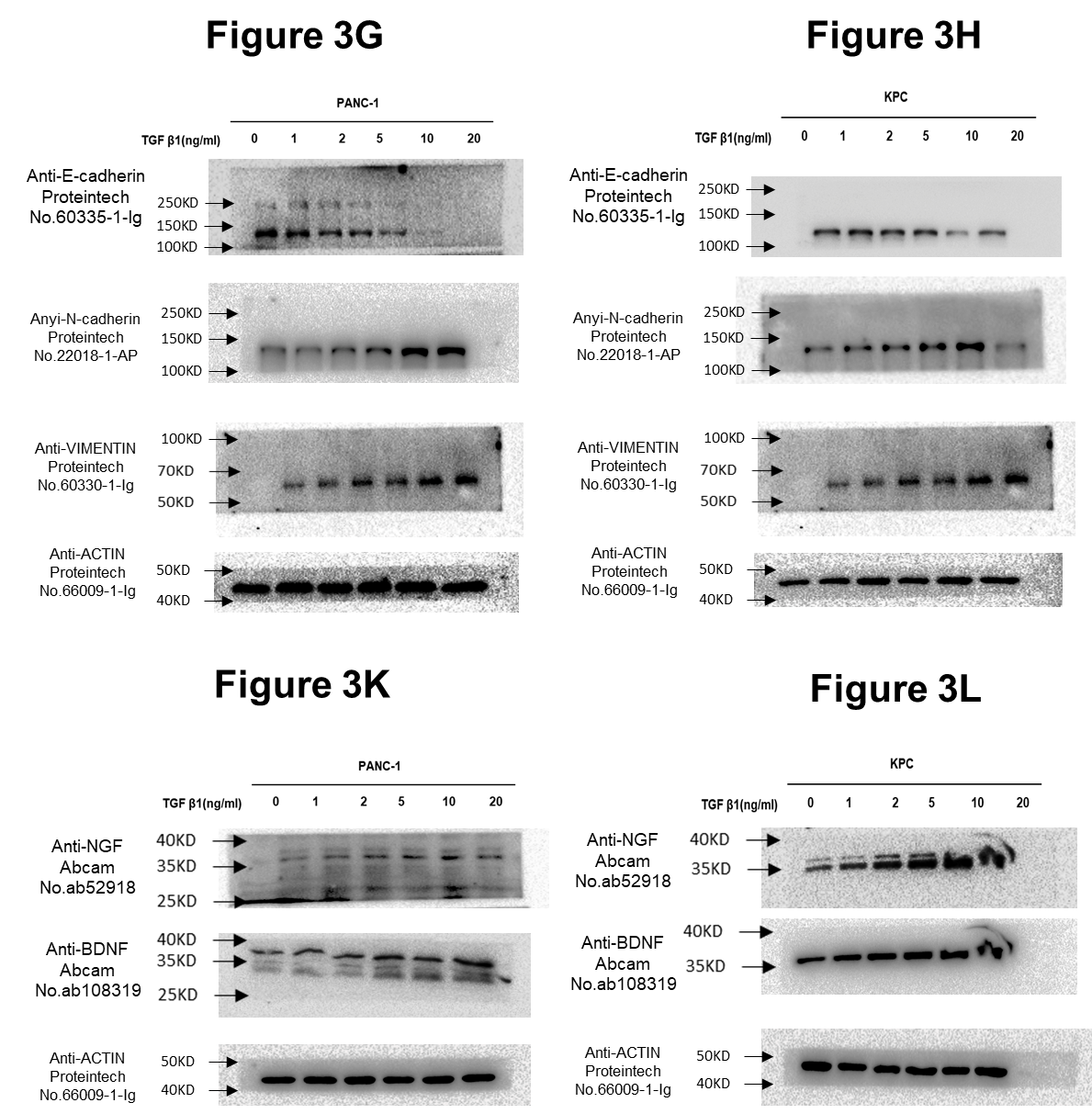


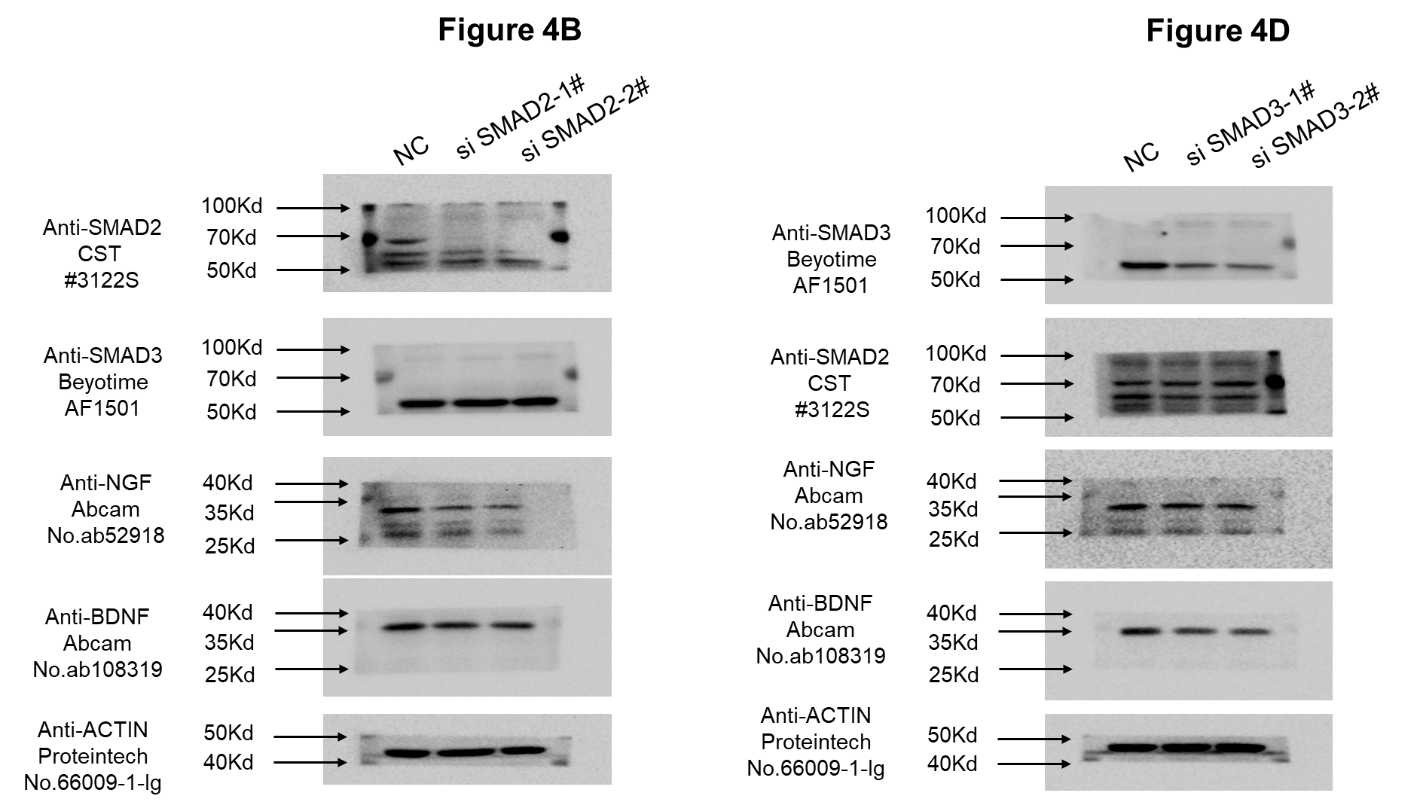


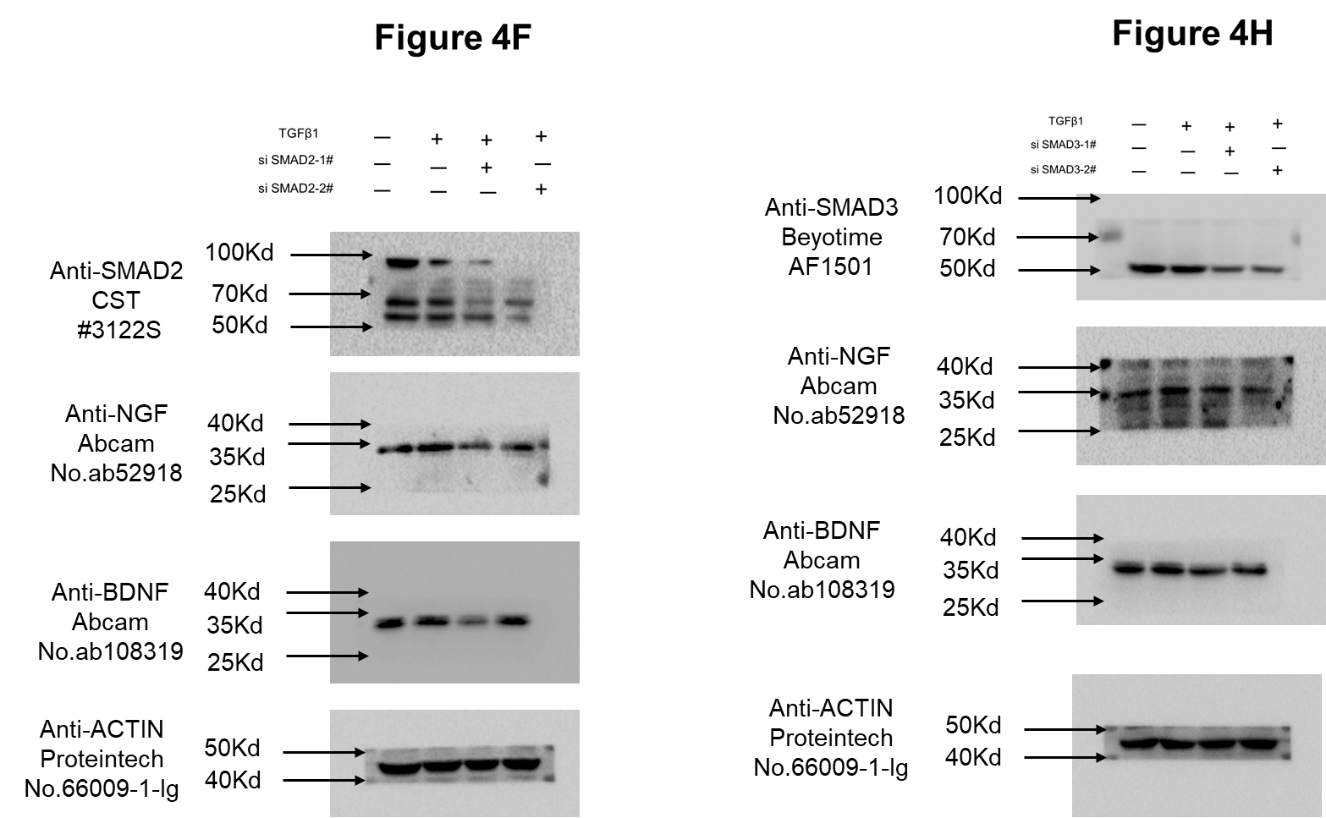


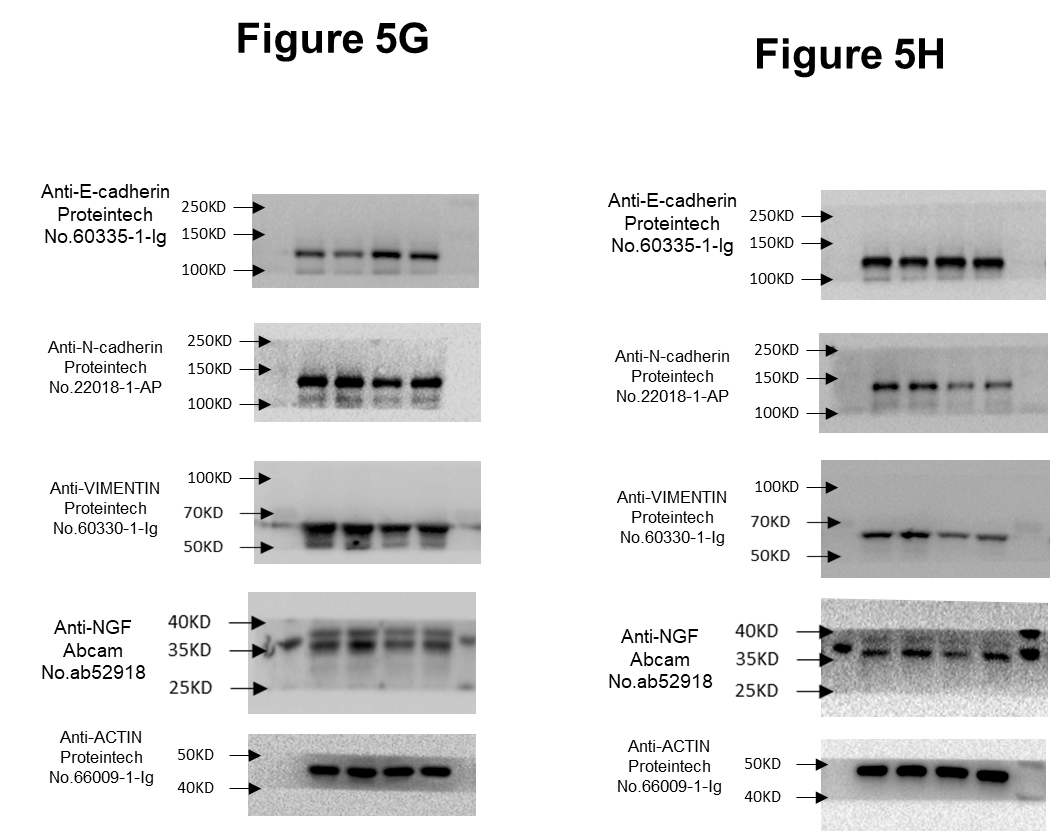


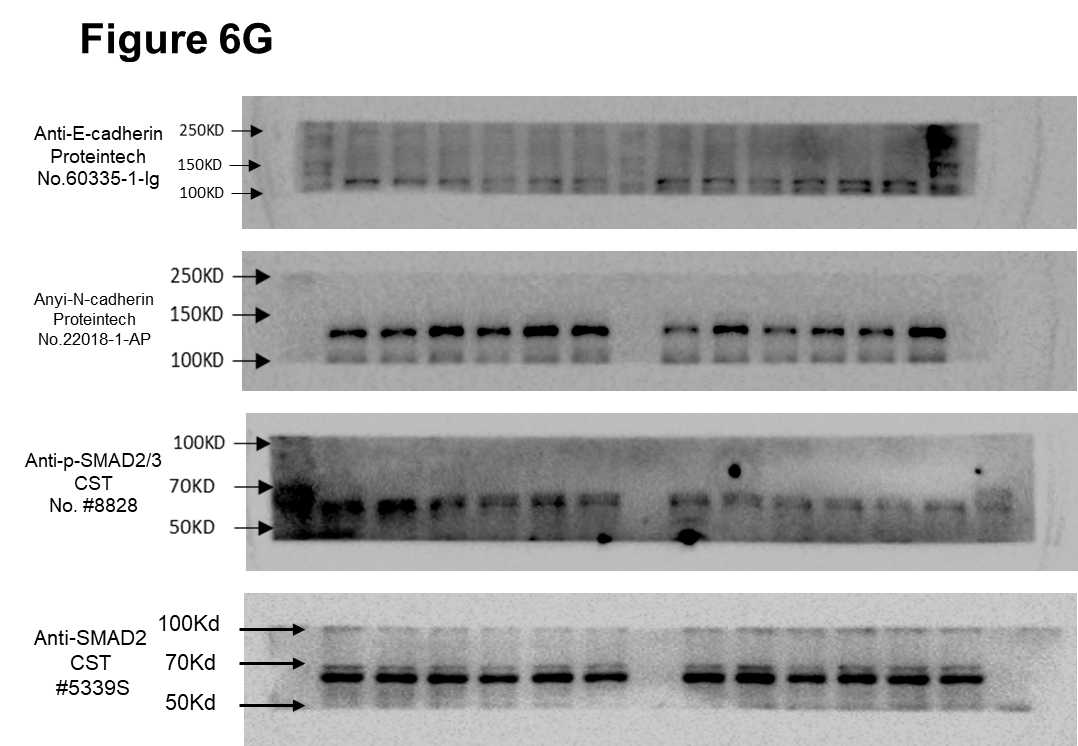

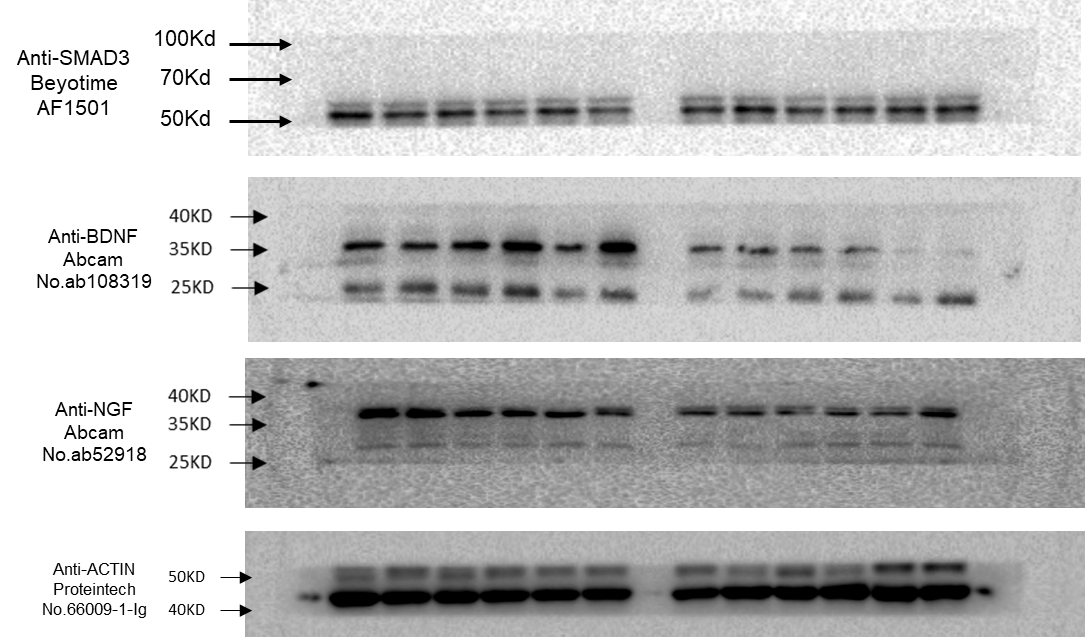

Supplement: Supplementary file 7 [file DataSheet_1.docx]
